# Differential Contributions of Five ABC Transporters to Mutidrug Resistance, Antioxidion and Virulence of *Beauveria bassiana*, an Entomopathogenic Fungus

**DOI:** 10.1371/journal.pone.0062179

**Published:** 2013-04-15

**Authors:** Ting-Ting Song, Jing Zhao, Sheng-Hua Ying, Ming-Guang Feng

**Affiliations:** 1 Institute of Microbiology, College of Life Science, Zhejiang University, Hangzhou, Zhejiang, People's Republic of China; 2 Horticulture Institute, Zhejiang Academy of Agricultural Sciences, Hangzhou, Zhejiang, People's Republic of China; University of Groningen, Netherlands

## Abstract

Multidrug resistance (MDR) confers agrochemical compatibility to fungal cells-based mycoinsecticdes but mechanisms involved in MDR remain poorly understood for entomopathogenic fungi, which have been widely applied as biocontrol agents against arthropod pests. Here we characterized the functions of five ATP-binding cassette (ABC) transporters, which were classified to the subfamilies ABC-B (Mdr1), ABC-C (Mrp1) and ABC-G (Pdr1, Pdr2 and Pdr5) and selected from 54 full-size ABC proteins of *Beauveria bassiana* based on their main domain architecture, membrane topology and transcriptional responses to three antifungal inducers. Disruption of each transporter gene resulted in significant reduction in resistance to four to six of eight fungicides or antifungal drugs tested due to their differences in structure and function. Compared with wild-type and complemented (control) strains, disruption mutants of all the five transporter genes became significantly less tolerant to the oxidants menadione and H_2_O_2_ based on 22−41% and 10−31% reductions of their effective concentrations required for the suppression of 50% colony growth at 25°C. Under a standardized spray, the killing actions of *ΔPdr5* and *ΔMrp1* mutants against *Spodoptera litura* second-instar larvae were delayed by 59% and 33% respectively. However, no significant virulence change was observed in three other delta mutants. Taken together, the examined five ABC transporters contribute differentially to not only the fungal MDR but antioxidant capability, a phenotype rarely associated with ABC efflux pumps in previous reports; at least some of them are required for the full virulence of *B. bassiana*, thereby affecting the fungal biocontrol potential. Our results indicate that ABC pump-dependent MDR mechanisms exist in entomopathogenic fungi as do in yeasts and human and plant pathogenic fungi.

## Introduction

Multidrug resistance (MDR) is a major challenge for the control of human, animal and plant pathogenic fungi by antifungal drugs and fungicides [1], [2], [3] but could be a merit for fungal entomopathogens against arthropod pests [4], [5]. This is because fungal cells, such as conidia produced on solid substrates, are the active ingredients of numerous mycoinsecticids and mycoacaricides [6] and MDR may confer their compatibility with chemical fungicides, herbicides and insecticides. Fungal candidate strains with higher MDR are more tolerant to applied chemical pesticides and thus more potential for commercial development and application.

MDR mechanisms in entomopathogenic fungi remain poorly understood although their compatibility with chemical pesticides has been emphasized as one of the determinants to a success of microbial control [7], [8]. Previously, some of common β-tubulin point mutations that are attributed to benzimidazole resistance in phytopathogenic fungi [9], [10] were found in *Beauveria bassiana* mutants with extraordinarily high carbendazim resistance [11]. However, none of such point mutations was found in *Isaria fumosorosea* mutants that showed not only as high carbendazim resistance as in the *B. bassiana* mutants but also resistance to other compounds different in structure and function [12]. Interestingly, all the *I. fumosorosea* mutants had three common point mutations occurred at the binding sites of the transcription factors Gal4, Abf1 and Raf in the promoter region of an ATP-binding cassette (ABC) transporter gene (*ifT1*) and thus their *ifT1* transcripts were upregulated by 17- to 137-fold. This implies that ABC transporter-dependent MDR mechanism exists in the fungal entomopathogens.

As a large family, ABC transporter proteins can energize the transport of a huge variety of compounds across biological membranes through ATP hydrolysis and confer cellular resistance to a broad spectrum of drug substrates [i.e., MDR or PDR (pleiotropic drug resistance) phenomenon] or a very limited number of substrates [13]. They are structurally featured with essential nucleotide-binding domain(s) (NBD) and one or two hydrophobic transmembrane domains (TMDs) and usually composed of six K-helical transmembrane segments (TMSs), forming the domain architectures of full-size [(TMS_6_−NBD)_2_ or (NBD−TMS_6_)_2_], half-size (TMS_6_−NBD) and TMD-lacking (NBD or NBD_2_) transporters [14]. Those associated with MDR/PDR are all full-size members classified to the subfamilies ABC-B (MDR type), ABC-C (MRP type, i.e., multidrug resistance-associated proteins) and ABC-G (PDR type) [15]. In human and plant pathogens, MDR/PDR results from drug efflux pumped by ABC transporters to reduce intracellular drug accumulation to toxic level at target sites [16], [17]. For instance, two PDR-type transporters, Cdr1p and Cdr2p, contribute differentially to azole resistance in *Candida albicans* [18], [19] due to their structural differences associated with substrate specificities and transport mechanism [20], [21]. ABC transporters also mediate cellular tolerance to natural toxic compounds and xenobiotics and/or virulence in many phytopathogenic fungi [22], [23], [24], [25]. Interestingly, the coding gene of a PDR-type ABC transporter in wheat supports durable resistance to wheat pathogenic fungi [26].

To explore possible MDR mechanisms in *B. bassiana*, we characterized the functions of three types of five representative proteins, which were selected from all full-size ABC transporter proteins by analyzing their phylogenetic and structural features and assessing their expressional responses to three different antifungal drugs. We found that the five transporters made differential contributions to the fungal MDR, antioxidation and virulence by multi-phenotypic comparisons of their single-gene disruption mutants with wild-type and complement strains

## Results

### Features of ABC transporters in *B. bassiana*

**Figure 1:**
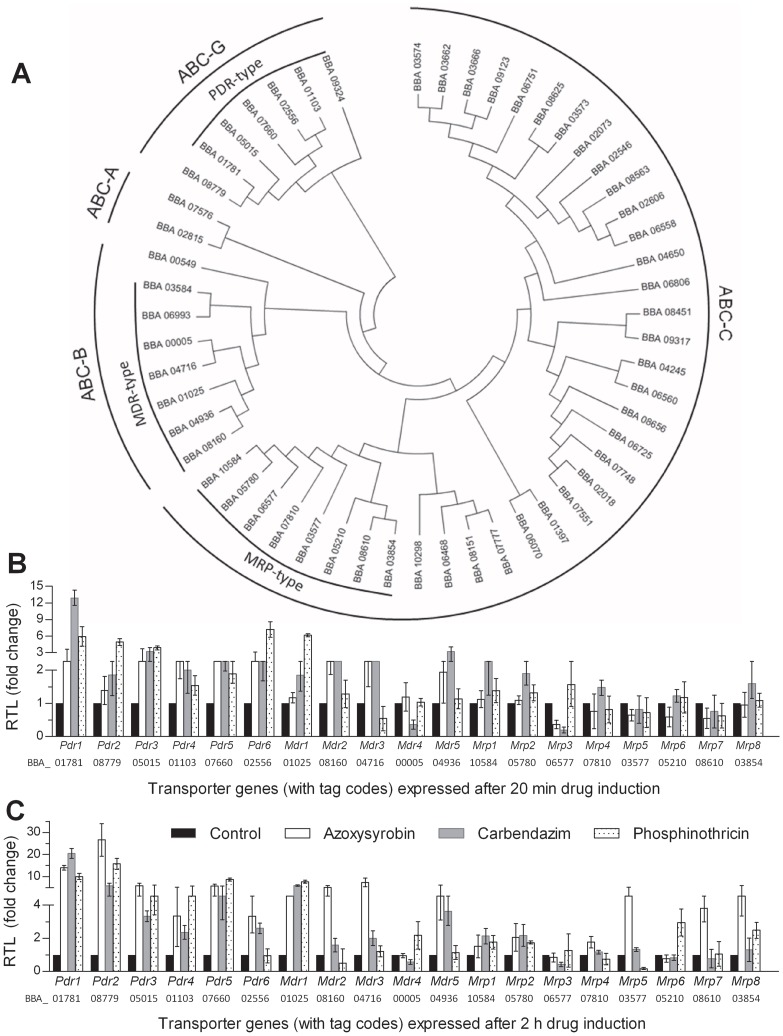
Screening of full-size ABC transporter proteins associated with multidrug resistance in *B. bassiana* (Bb2860). (A) Phylogenetic analysis of 54 full-size ABC proteins. (B), (C) Relative transcript levels (RTL) of 21 ABC transporter genes in the wild-type SDB cultures induced with carbendazim (5 µg/ml), azoxysyrobin (100 µg/ml) and phosphinothricin (100 µg/ml) for 20 min and 2 h at 25°C respectively. Error bars: SD of the mean from three cDNA samples assessed via qRT-PCR with paired primers (Table S1).

Up to 425 transporter proteins were blasted from the annotated genome of the wild-type strain *B. bassiana* ARSEF 2860 (Bb2860 or wild type herein) [27], including 54 putative ABC pumps coupled with the queries of conserved NBD and TMD regions of budding yeast Ste6p, Pdr5p or Yor1p in the NCBI protein database. The 54 proteins were classified to four ABC subfamilies (Fig. 1A). The largest ABC-C subfamily includes 37 members, of which eight are likely MRP-type transporters based on their membrane topology. Further comparison of domain architecture led to the recognition of seven ABC-B and six ABC-G proteins as potential MDR- and PDR-type transporters respectively. All the 21 recognized transporters of three types are featured with two NBDs and two TMDs

The 21 ABC transporters were assessed for the levels of their gene transcripts in wild-type hyphal cells induced with azoxysyrobin, carbendazim and phosphinothricin for 20 min and 2 h at 25°C respectively. As a result of quantitative real-time PCR (qRT-PCR) with paired primers (Table S1), about half of them were upregulated by the drug inducers within 20 min (Fig. 1B). Longer induction enhanced their transcripts to higher levels (Fig. 1C). Consequently, Pdr1, Pdr2, Pdr5, Mdr1 and Mrp1 were chosen as the representatives of the three types because they were inductively upregulated by all the three drugs. Notably, *Mdr6* and *Mdr7* transcripts were consistently undetectable in the cDNAs from the samples induced or not induced with the drugs (data not shown).

The coding genes of the selected five transporters were disrupted from Bb2860 and complemented into their disruption mutants by integration of the *bar*- and *sur*-inclusive plasmids via *Agrobacterium*-mediated transformation respectively. Putative mutant colonies grown on selective plates were sequentially identified via PCR, reverse transcription PCR (RT-PCR) and Southern blotting with paired primers and amplified probes (Table S2). As a result of the identification, the profiling band or signal for each target gene was consistently present in the wild-type and complement strains (control strains) but absent in the disruption mutant (Fig. S1). Thus, five single-gene disruption mutants were compared with the control strains to differentiate their phenotypic changes below.

### Differentiated MDR responses

**Figure 2:**
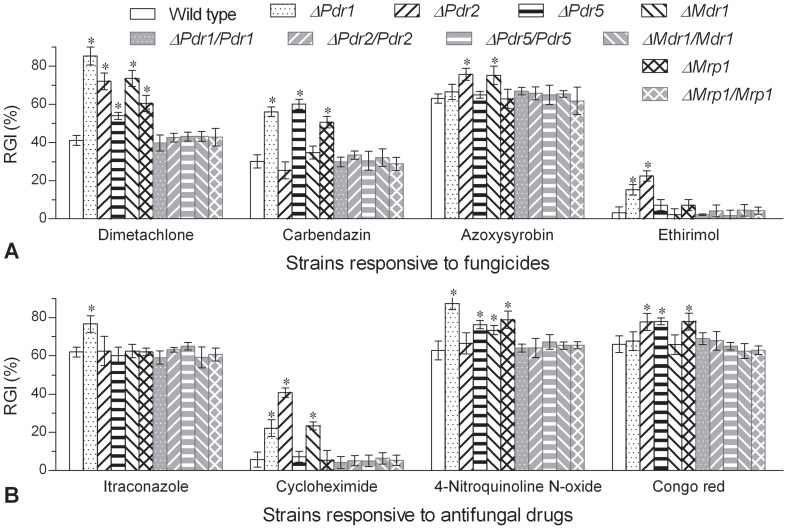
Changes in multidrug resistance of five single-gene disruption mutants of *B. bassiana*. (A) Relative growth inhibition (RGI) of fungal colonies after 6-day incubation at 25°C on 1/4 SDAY supplemented with the fungicides dimetachlone (0.1 mg/ml), carbendazim (0.5 µg/ml), azoxysyrobin (0.1 mg/ml) and ethirimol (1 mg/ml) respectively. (B) RGI values of fungal colonies after 6-day incubation at 25°C on 1/4 SDAY supplemented with the antifungal drugs itraconazole (5 µg/ml), cyclonheximide (20 µg/ml), 4-nitroquinoline-N-oxide (5 µg/ml) and Congo red (0.5 mg/ml) respectively. The bars of each group marked with asterisks differed significantly from those unmarked (Tukey's HSD, *P*<0.05). Error bars: SD of the mean from three repeated assays.

All the five disruption mutants showed differential resistance to different types of four fungicides (Fig. 2A) and four antifungal drugs (Fig. 2B) during 6-day growth on 1/4 SDAY at 25°C but their control strains responded equally to each drug (Tukey's HSD, *P*>0.1). Compared with the means of relative growth inhibition (RGI) values observed in the control strains, six, five and four of the tested chemicals were significantly more inhibitory to *ΔPdr1* (12−43%), *ΔPdr2* (11−35%) and three other delta mutants (9−31%), respectively. During the colony growth, dimetachlone exerted inhibitory effect on all the delta mutants while itraconazole, azoxysyrobin and ethirimol were influential only on one or two of them. Null responses were observed in *ΔPdr1* to Congo red and azoxysyrobin and in *ΔPdr2* to carbendazim, itraconazole and 4-nitroquinoline-N-oxide. These data indicated that the spectra and preference of drug substrates were partially different among the five ABC transporters.

Additionally, all the disruption mutants and the control strains grew equally well on drug-free SDAY or 1/4 SDAY at 25°C (*P*>0.15 in *F* tests) and responded equally to hyperosmotic (NaCl) stress (*F*_10,22_ = 1.25, *P* = 0.32; data not shown).

### Differentiated antioxidation responses

**Figure 3:**
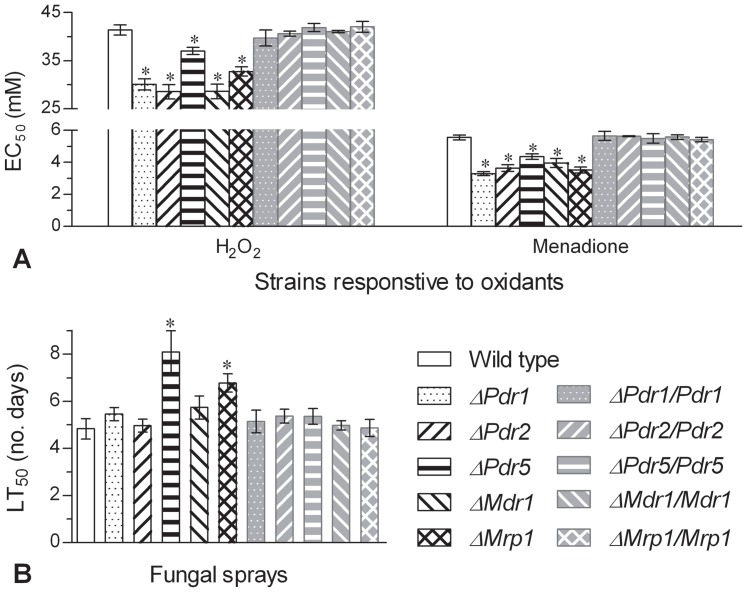
Changes in antioxidant capability and virulence of five single-gene disruption mutants of *B. bassiana*. (A) Effective concentrations (EC_50_s) estimated for H_2_O_2_ and menadione to suppress 50% colony growth by modeling analysis of relative growth trends over the concentrations of 0−80 mM H_2_O_2_ or 0−8 mM menadione added to 1/4 SDAY. (B) Median lethal times (LT_50_s) of wild-type and mutant strains against the second-instar larvae of *S. litura* under a standardized spray. The bars of each group marked with asterisks differed significantly from those unmarked (Tukey's HSD, *P*<0.05). Error bars: SD of the mean from three repeated assays.

Two oxidants, H_2_O_2_ and menadione, were assayed for their effective concentrations (EC_50_s) to suppress 50% colony growth of each strain by modeling analysis of relative growth trend over the gradient concentrations of each oxidant after 6-day incubation at 25°C. Compared with the EC_50_ estimates of menadione [5.6 (±0.09) mM] and H_2_O_2_ [41.1 (±0.87) mM] towards the control strains (Fig. 3A), all the five delta mutants were 22−41% less tolerant to menadione and 10−31% less tolerant to H_2_O_2_ (Tukey's HSD, *P*<0.01).

### Differentiated virulence

Time-mortality trends of all the tested strains against the second-instar larvae of *Spodoptera litura* in the bioassays standardized by a uniform spray of conidial suspension in an automatic spray tower were differentiated by probit analysis. Median lethal time (LT_50_) estimates fell in a narrow range of 4.8−5.3 (average 5.1) days for all the control strains (Tukey's HSD, *P*≥0.15) but significantly increased to 8.1 and 6.8 days for *ΔPdr5* and *ΔMrp1* respectively (Fig. 3D). However, no significant LT_50_ differences were found between other delta mutants and the control strains.

## Discussion

*B. bassiana* harbors 21 full-size ABC transporter genes that may act as potential MDR regulators due to their classification, membrane topology and domain architecture. Of those, however, *Mdr6* and *Mdr7* had no detectable transcriptional signals in the cDNAs from the total RNAs of the wild-type cultures induced with drugs or not induced, suggesting a likelihood of their pseudogene status. Five representatives selected by their transcript levels inductively upregulated by azoxysyrobin, carbendazim and phosphinothricin were confirmed contributing differentially to the fungal MDR, antioxidation and virulence but not involving in osmoregulation, as discussed below.

First of all, up to 425 transporter proteins can be blasted from the genome of *B. bassiana* [27] and the counts of the counterparts in the genomes of *Metarhizium robertsii* (previously in *M anisopliae* sensu lato) and *M. acridum* [28] are 304 and 307 respectively. Despite a remarkable diversity, only a small proportion of them are full-size ABC pumps of *B. bassiana* (Fig. 1A) and an even smaller proportion are likely associated with the fungal MDR/PDR in terms of their membrane topology and main domain architecture (14,15,21). The five transporters more responsive to the three inducers (Fig. 1) were all proven to regulate MDR in *B. bassiana* because their single-gene disruption mutants showed less resistance to four to six of the eight antifungal drugs (Fig. 2), which were used in MDR assays due to differences in structure and function. Apparently, ABC pump-dependent MDR mechanisms exist in entomopathogenic fungi as do in yeasts and human and plant pathogenic fungi [13], [16].

Despite partially overlapping drug spectra, the examined five transporters showed some degree of substrate preference based on the MDR changes in their delta mutants. The preferred substrate was dimetachlone for Pdr1 and Mdr1, carbendazim for Pdr5 and Mrp1, and cycloheximide for Pdr2. As a broad-spectrum fungicide, dimetachlone was a mere common substrate for the five transporters but itraconazole was specific to only Pdr1 among the tested drugs. Moreover, the drug spectrum was broadest for Pdr1, followed by Pdr2 and three others. Regardless of broader or narrower substrate spectrum, Pdr1, Pdr2, Pdr5, Mdr1 and Mrp1 are functionally very close to other fungal ABC transporters, such as those mediating *Aspergillus nidulans* resistance to all major classes of fungicides [29], *Botrytis cinerea* sensitivity to phenylpyrrole fungicides [30], and *Mycosphaerella graminicola* responses to azole fungicides [25]. Their drug preferences, substrate spectra and MDR levels altered by single-gene disruption are partially different from one to another. This is in accordance with those of documented fungal ABC pumps between different types [13] or within a type [18], [19] and likely due to low primary sequence similarity between their TMDs [21]. Thus, the five transporters of *B. bassiana* regulate differentially the fungal MDR/PDR.

Apart from differential responses to the tested antifungal drugs, all five delta mutants showed significantly less, but differential, resistance to the oxidants menadione and H_2_O_2_ (Fig. 3A). Fungal antioxidant capability has rarely been associated with ABC transporters in previous studies but is important for the success of *B. bassiana* infection. This capability usually depends on the activities of antioxidative enzymes, such as catalases [31] and superoxide dismutases [32], [33], and can be regulated by cellular signaling pathways, such as the mitogen-activated protein kinase cascades of Hog1 [34] and Slt2 [35], P-type calcium ATPase [36] and Ras1/Ras2 GTPases [37]. Particularly, fungal tolerance to oxidation is linearly correlated with *B. bassiana* UV resistance and virulence [33], [37], two parameters important for the fungal biocontrol potential. Thus, the antioxidant capability reduced by the disruption of each ABC transporter gene implies that the fungal pathogen is less capable of scavenging harmful superoxide anions often generated from infected host cells. We consider that the five transporters could pump both oxidants as they usually pump xenobiotic efflux although they are not antioxidant enzymes.

Finally, fungal virulence has been infrequently associated with the effects of ABC transporters but this association has been found in some phytopathogenic fungi. For instance, three ABC pumps, namely ABC1 in *Magnaporthe grisea* [24], NhABC1 in *Nectria haematococca* [22] and BcatrB in *Botrytis cinerea* [38], have proved to influence the fungal virulence due to their pumping action of cytotoxic compound efflux. In this study, only Pdr5 and Mrp1 were found contributing significantly to the virulence of *B. bassiana* to *S. litura* larvae because the killing actions of their delta mutants under a standardized spray were 59% and 33% slower than those of the control strains (Fig. 3B). However, three other transporters we examined showed null effect on the fungal virulence. Taken together with previous reports and our results, not all ABC transporters are contributors to fungal virulence but at least some of them are necessary for the full virulence of a fungal pathogen, thereby affecting the biocontrol potential of *B. bassiana*.

## Materials and Methods

### Microbial strains and culture conditions

The wild-type strain Bb2860 was cultured on Sabouraud dextrose agar plus 1% yeast extract (SDAY) at 25°C and used as a recipient of gene manipulation and expression. *Escherichia coli* Top10 and *E. coli* DH5α from Invitrogen (Shanghai, China) used for vector propagation were cultured at 37°C in LB medium plus kanamycin (100 µg/ml). For fungal transformation, *A. tumefaciens* AGL-1 was cultured in YEB medium [39] at 28°C.

### Phylogenetic, structural and transcriptional analyses of *B. bassiana* ABC transporters

The conserved NBD and TMD regions of the typical ABC transporters Ste6p, Pdr5p and Yor1p in *Saccharomyces cerevisiae* (NCBI accession codes: NC_001143.9, NC_00147.6 and NC_00113.9 respectively) were used as queries to locate PDR-, MDR- and MRP-type transporters respectively in the sequenced genome of Bb2860 under the NCBI accession ADAH00000000 [27] via blastp (http://blast.ncbi.nlm.nih.gov/blast.cgi) and BioEdit analysis (http://www.mbio.ncsu.edu/bioedit/bioedit.html). The resultant protein sequences were screened to remove half-size transporters not associated with MDR in terms of their domain architecture and then classified to ABC subfamilies following a documented system [15] via phylogenetic analysis with MEGA 4.0 software [40]. Their membrane topological features were further analyzed to assess the likelihood of their involvements in fungal MDR/PDR [13] via CD search (http://www.ncbi.nlm.nih.gov/Structure/cdd/wrpsb.cgi), generating 21 full-size ABC transporters (Mdr1−7, Mrp1−8 and Pdr1−6) for study.

To assess the transcriptional expression levels of the selected transporters in response to different antifungal chemicals, Bb2860 was grown in 50 ml aliquots of Sabouraud dextrose broth (SDB) inoculated to 1×10^6^ conidia/ml. After 2-day shaking by 120 rpm at 25°C, hyphal cells were harvested from the cultures and transferred to the same volume of fresh SDB supplemented with carbendazim (benzimidazole fungicide, 5 µg/ml), azoxysyrobin (broad-spectrum fungicide, 100 µg/ml) or phosphinothricin (herbicide, 100 µg/ml) for the induction of 20 min and 2 h at 25°C respectively. Total RNAs were extracted from the drug-induced and drug-free (control) cultures. Three samples of 5 µg RNA from each extract was reversely transcribed with PrimeScript™ RT kit (Takara, Dalian, China). The cDNA samples (diluted to 10 µg/ml) synthesized with the kit were assessed for the transcript levels of all the 21 transporter genes via qRT-PCR with paired primers (Table S1) using *B. bassiana* 18S rRNA as internal standard. The relative transcript level of each gene in a drug-induced sample versus control was calculated as its transcript ratio using the method 2^−ΔΔCt^ [41]. Five transporter genes whose transcript levels were inductively upregulated by all the three drugs were selected for further study, including *Pdr1*, *Pdr2*, *Pdr5*, *Mdr1* and *Mrp1*.

### Single-gene disruption and complementation

The plasmid p0380-bar vectoring the *bar* marker and P*trpC* promoter [33] was used as backbone to construct the disruption plasmids of all selected genes except *Pdr1*. The 5′ and 3′ fragments of *Pdr2* (1261 and 1874 bp), *Pdr5* (1560 and 1666 bp), *Mdr1* (1480 and 1147 bp) and *Mrp1* (1531 and 1320 bp) were separately amplified from Bb2860 via PCR with paired primers (Table S2), digested with specific restriction enzymes, and inserted into p0380-bar, generating p0380-5′*x*-bar-3′*x* for the disruption of each target gene (*x*). To delete *Pdr1*, alternatively, its ORF fragment (2500 bp) was amplified from Bb2860 with Pdr1-F/R (Table S2) and inserted into p0380-bar linearized with *Bam*HI/*Hin*dIII. After digestion with *Xho*I/*Sac*I, the plasmid released a fragment of ∼200 bp to separate the ORF into two fragments and a P*trpC*-*bar* cassette amplified from p0380-bar with the Insert-F/R primers was inserted between the separated fragments, yielding p0380-pdr1D for *Pdr1* disruption.

To rescue each of the disrupted genes, p0380-sur-gateway [33] vectoring the *sur* marker gene was used as backbone. The full-length sequences with flanking regions of *Pdr1* (7899 bp), *Pdr2* (7926 bp), *Pdr5* (7754 bp), *Mdr1* (7850 bp) and *Mrp1* (7389 bp) were separately amplified from Bb2860 with paired primers (Table S1) under the action of LA*Taq* polymerase (TaKaRa) and ligated into the backbone to replace the gateway fragment under the action of Gateway*®* BP ClonaseTM II Enzyme Mix (Invitrogen), forming p0380-sur-*x*, where *x* denotes one of the rescued target genes.

All the disruption and complement plasmids were individually transformed into *A. tumefaciens* AGL-1 for further transformation into Bb2860 or the corresponding disruption mutants using a documented protocol [39] with slight modification. Briefly, the recipient strain (wild type or delta mutant) was co-cultivated with the vector-integrated AGL-1 on induced medium for 48 h at 25°C in dark, followed by washing with ∼5 ml of 0.02% Tween 80. The suspension was spread onto M-100 plates [39] supplemented with cefotaxime (300 µg/ml for suppressing AGL-1 growth) and phosphinothricin (200 µg/ml for the selective growth of disruption mutants) or chorimuron ethyl (10 µg/ml for the selective growth of rescued mutants). All the plates were incubated for 6 days at 25°C and 12∶12 h (light:dark cycle). Colonies grown on the selective plates were identified via PCR, RT-PCR and Southern blotting with paired primers or amplified probes (Table S2). For Southern blotting, 30 µg genomic DNA extracted from the monoclonal culture of each putative mutant on SDAY was digested with *Xba*I/*Hin*dIII, separated via electrophoresis in 0.7% agarose gel, and then transferred to Biodyne B nylon membrane (Gelman Laboratory, Shelton, WA, USA) in Trans-Blot SD Electrophoretic Transfer Cell (Bio-Rad, Hercules, CA, USA). Probe preparation, membrane hybridization and visualization were carried out using DIG High Prime DNA Labeling and Detection Starter Kit II (Roche, Mannheim, Germany). Positive disruption and complement mutants of each target gene were assayed together with wild type for their phenotypic changes in triplicate experiments below.

### Assays of multidrug responses

The aliquots of 200 µl conidial suspension (2×10^7^ conidia/ml) were evenly spread onto cellophane-overlaid SDAY plates. After 3-day incubation at 25°C and 12∶12 h, cellophane discs (5 mm diameter) with growing mycelia were cut from the culture of each strain and attached centrally onto the plates (9 cm diameter) of 1/4 SDAY (SDAY nutrients diluted to 1/4) supplemented with the antifungal drugs azoxysyrobin (100 µg/ml), carbendazim (0.5 µg/ml), dimetachlone (pyrrole fungicide, 100 µg/ml), ethirimol (pyrimidine fungicide, 1 mg/ml), itraconazole (triazole agent, 5 µg/ml), cyclonheximide (protein biosynthesis inhibitor, 20 µg/ml), 4-Nitroquinoline N-oxide (potent mutagenic agent, 10 µg/ml) and Congo red (cell wall biosynthesis inhibitor, 500 µg/ml) for MDR assays and NaCl (40 mg/ml) for osmosensitivity assay respectively. All the plates were incubated for 6 days at the same regime, followed by cross-measuring the diameters of their colonies. For each strain stressed with a given drug, relative growth inhibition (RGI) was calculated as (*C*–*N*)/(*C*–19.6)×100, where the constant is the area of inoculated disc, and *C* and *N* denote the measurements of colony area (mm^2^) from the control (free of drug) and drug treatment respectively.

To quantify antioxidant capability of each strain, the plates of the same medium supplemented with the gradient concentrations of menadione (0−8 mM) or H_2_O_2_ (0−80 mM) for varying intensity of oxidative stress were inoculated with the culture discs as above. After 6-day incubation at the same regime, colony diameters were cross-measured and the ratio of a stressed colony size over the size of the control colony was defined as relative growth rate (*R*_g_). The *R*_g_ trend of each strain over the concentrations (*C*) of menadione or H_2_O_2_ was fitted to the equation *R*_g_ = 1/[1−exp(*a*+*bC*)]. Solving the fitted equation gave an effective concentration of each oxidant (EC_50_) to suppress 50% colony growth when *R*_g_ = 0.5.

### Virulence bioassay

All the fungal strains were bioassayed for their changes in virulence to the second-instar larvae of *S. litura* using a standardized method described elsewhere (36,37). Briefly, batches of 30−40 larvae on cabbage leaf discs (∼10 cm diameter) were separately sprayed with 1 ml of conidial suspension (2×10^7^ conidia/ml) as treatment or 0.02% Tween 80 (used for suspending conidia) as control in automatic Potter Spray Tower (Burkard Scientific Ltd, Uxbridge, UK). After spray, all larvae were reared on the leaf discs in large Petri dishes (15 cm diameter) for 7 days at 25°C and 12∶12 h and fresh leaf discs were supplied daily for their feeding. Mortality in each plate was daily examined during the period. The resultant time-mortality trends were subjected to probit analysis, generating an estimate of medial lethal time (LT_50_) for each fungal strain against the pest species.

## Supporting Information

Table S1 **Paired primers used for assessing the transcript levels of 21 full-size ABC transporter genes in** ***B. bassiana*** **via qRT-PCR.**
(DOC)

Table S2 **Paired primers used for the manipulation of five ABC transporter genes in** ***B. bassiana*** **.**
(DOC)

Figure S1 **Disruption and complementation of five selected ABC transporter genes in** ***B. bassiana*** **wild-type strain (Bb2860).**
(JPG)
