# Supplementary material for: Differential Contributions of Five ABC Transporters to Mutidrug Resistance, Antioxidion and Virulence of Beauveria bassiana, an Entomopathogenic Fungus
Source: PLoS One. 2013 Apr 15;8(4):e62179. doi: 10.1371/journal.pone.0062179 (PMC3626590; doi:10.1371/journal.pone.0062179)
Supplement: Table S1 — Paired primers used for assessing the transcript levels of 21 full-size ABC transporter genes in B. bassiana via qRT-PCR. (DOC) [file pone.0062179.s001.doc]

**Table S1.** Paired primers used for assessing the transcript levels of 21 full-size ABC transporter genes in *B. bassiana* via qRT-PCR.

| Gene | Gene ID* | Sequences of paired primers (5′-3′) |
| --- | --- | --- |
| 18S RNA |  | TCGGTTTCTAGGACCGCCGTAA / CCTTGGCAAATGCTTTCGC |
| *Pdr1* | BBA_01781 | CACTGCTAGATTGCTTGGCTGACC / ACCGTCGTTGTCTGGAGATGGAG |
| *Pdr2* | BBA_08779 | CGACGAGACGCAGGTTCATTCTTC / GACAGCCGAAGGAGCCAATGC |
| *Pdr3* | BBA_05015 | GCGAGCCTGCCCAATTCTTTCTG / GCTTGTGAGACGGTCCTGGTAGAG |
| *Pdr4* | BBA_01103 | GCCAGTTCATTGCCGCCTACG / AAGGAGACGAGGACACCGATGATG |
| *Pdr5* | BBA_07660 | TCCTGCCCTTCTTCCTCGTCATG / AGAGCACGCCGCCGACATAG |
| *Pdr6* | BBA_02556 | GCCTCACCGTCTTCTTCACCAATG / GCTGTGCCTGCTCCTCATCTTTG |
| *Mdr1* | BBA_01025 | TGCTGCCTGTGCCGTCTCC / TGGACCGCCGCTGGAAGTG |
| *Mdr2* | BBA_08160 | CACGCCGCCAAGGTCGCCAACTG / CGCTGCTTCTGCCCGCCCGAGAG |
| *Mdr3* | BBA_04716 | GCGGTCTTGTCGGTCCCTCTG / GCGGCACCACGGCAATGTC |
| *Mdr4* | BBA_06993 | CGCTTCTACGACCCGACCTCTG / TGCTGGCACGCCGAGATAACC |
| *Mdr5* | BBA_03584 | GCTCGCTGCCAACAAGGACAGTG / TCTTCGCCCTGCTCCCCTTCAATG |
| *Mdr6* | BBA_00005 | AGGAAGAGCCAGCATATC / ATAGCGAGAGCCATCATC |
| *Mdr7* | BBA_04936 | CTTCATCGCCATCATCTTC / TTCAATGTCGCTGTCGTA |
| *Mrp1* | BBA_10584 | TCTTGGTTGGGCATTCGGCTTGG / TTGCTGAGTATGGTCGCTGCTGTC |
| *Mrp2* | BBA_05780 | GCAAGATTAACCTTCTCACAA / CATCATAACGAACAGTCACAT |
| *Mrp3* | BBA_06577 | GAGCAGCAGCATTGTATC / GTGAGATGGCAAGTTGATG |
| *Mrp4* | BBA_07810 | CGGCTTACACGCAGAGTT / GCTCGGCTTCTCCAATCAG |
| *Mrp5* | BBA_03577 | GCCACCATAAGAGCATTC / ACCAACGACTGAAGATGTA |
| *Mrp6* | BBA_05210 | TATGATAGCGTGGTGGTTAT / TTGAGAATCGTCTGGAGTT |
| *Mrp7* | BBA_08610 | ATCAGCAACATCATCCAATC / AGCAGCCAATAGTTAGCA |
| *Mrp8* | BBA_03854 | GAATGTTACCGTGCGATAC / CCAGATATTATGTGCGTGAAT |

* Tag codes in the Bb2860 genome under the NCBI accession ADAH00000000 [27]
