# Supplementary material for: Differential Contributions of Five ABC Transporters to Mutidrug Resistance, Antioxidion and Virulence of Beauveria bassiana, an Entomopathogenic Fungus
Source: PLoS One. 2013 Apr 15;8(4):e62179. doi: 10.1371/journal.pone.0062179 (PMC3626590; doi:10.1371/journal.pone.0062179)
Supplement: Table S2 — Paired primers used for the manipulation of five ABC transporter genes in B. bassiana . (DOC) [file pone.0062179.s002.doc]

**Table S2. Paired primers used for the manipulation of five ABC transporter genes in *B. bassiana*.**

| Primers | Paired sequences (5'−3')* | Purpose |
| --- | --- | --- |
| Pdr1-F/R | CGGGATCCGGTCCGCCTTTCGCACAG / CCCAAGCTTCGGCGTGTTTCGCTCTT | Cloning *Pdr1* ORF for deletion |
| Insert-F/R | CGAGCTCGAATTCGTCGACGTTAACTG / CCGCTCGAGTCAAATCTCGGTGACGGGC | Cloning an insert for *Pdr1* deletion |
| 5′Pdr2-F/R | CGGAATTCATCGGGGTGTTCGACCTTTCT / CGGGATCCGGTGGCAATGAGATAGACACTT | Cloning *Pdr2* 5′-end for deletion |
| 3′Pdr2-F/R | ATGCCATGGATCTTTCACTGGAACAATGTCTGC / AAGATCTCCAAAGAGTGTCAGAAGCATAAAGA | Cloning *Pdr2* 3′-end for deletion |
| 5′Pdr5-F/R | TCCCCCGGGCGACCTGATTCACGACTTTACCG / CCCAAGCTTCAGTAGAACCAACCGAACCAAAT | Cloning *Pdr5* 5′-end for deletion |
| 3′Pdr5-F/R | GAAGATCTGCTCAACAACGTGCAAGGTTAC / GACTAGTCATGTCGGTAGCATTCGGGTC | Cloning *Pdr5* 3′-end for deletion |
| 5′Mdr1-F/R | CGGAATTCAAGGCTTCGCTTGAATCTTACG / GGGGTACCAATATGATCGTCGGGGTCGG | Cloning *Mdr1* 5′-end for deletion |
| 3′Mdr1-F/R | CATGCCATGGTGTTCGTGACCGTGCATTTCG / GAAGATCTATCGGGCAGCGAGAT GATAAA | Cloning *Mdr1* 3′-end for deletion |
| 5′Mrp1-F/R | CGAGCTCCTCTTGTATTTGTTCCGCATCTTG / CGGGATCCCCAATGCAGAAGTTCGCTCAGTAG | Cloning *Mrp1* 5′-end for deletion |
| 3′Mrp1-F/R | CCGCTCGAGCCGACGAACAAGTCCACGAA / GAAGATCTCCAGAGTGGATAGTGCGGTCAAG | Cloning *Mrp1* 3′-end for deletion |
| flPdr1-F/R | *GGGGACAAGTTTGTACAAAAAAGCAGGCT*GGTTGTGGTCTCGTCCTATTTCTT / *GGGGACCACTTTGTACAAGAAAGCTGG GT*GGGCAGGGTATTCTGGATTGA | Cloning full-length *Pdr1* for rescuing |
| flPdr2-F/R | *GGGGACAAGTTTGTACAAAAAAGCAGGCT*CCTCGGTGCGTTGTTATCGG / *GGGGACCACTTTGTACAAGAAAGCTGGGT*TG CCGTTTGGAGACTTTGTGAA | Cloning full-length *Pdr2* for rescuing |
| flPdr5-F/R | *GGGGACAAGTTTGTACAAAAAAGCAGGCT*AGTCCGTGACAAACATCAAACATC / *GGGGACCACTTTGTACAAGAAAGCTGG GT*ACCTACTGGGTCTCCGTGCC | Cloning full-length *Pdr5* for rescuing |
| flMdr1-F/R | *GGGGACAAGTTTGTACAAAAAAGCAGGCT*GTTCGGCTCGGACACTTGGA / *GGGGACCACTTTGTACAAGAAAGCTGGGT*CA CCCACAAACCGTCCCAAT | Cloning full-length *Mdr1* for rescuing |
| flMrp1-F/R | *GGGGACAAGTTTGTACAAAAAAGCAGGCT*ACGAGACCTCAGGGTTAGGAAAG / *GGGGACCACTTTGTACAAGAAAGCTGG GT*AGCAAGATCCACTACCGAGGG | Cloning full-length *Mrp1* for rescuing |
| Bar-F/R | AGAACGACGCCCGGCCGACAT / CTGCCAGAAACCCACGTCATGC | Mutant ID by PCR for *bar* |
| pPdr1-F/R | GTCCGTGGTGTTTCTGGTGGT / GGATGGCTTGTCTTGTATTCCTCA | Mutant ID by PCR for *Pdr1* |
| pPdr2-F/R | TGCATCGCTGTCTCGGACTC / CGATTCTCCTTGCCGATTTTG | Mutant ID by PCR for *Pdr2* |
| pPdr5-F/R | ACTGTGGAGGGCGGCTATTG / CATCATTGGATACGCCAGAACAA | Mutant ID by PCR for *Pdr5* |
| pMdr1-F/R | ATGTCTCAAGGCAAAATCGTCG / CGAAGTATGGCCCGAAATGC | Mutant ID by PCR for *Mdr1* |
| pMrp1-F/R | GGGCTTCACAACTGTCATTATCG / GTGTTGGCTTTTGCGTGGAA | Mutant ID by PCR for *Mrp1* |
| rtPdr1-F/R | GGCAGCACAGCTTGCGTCTC / AGTGACGAGGTCATGGACGTCA | Mutant ID by RT-PCR for *Pdr1* |
| rtPdr2-F/R | CCGAGAAAAAGTCCAAAGGCG / TTTGGTGGGCAATCATGGCA | Mutant ID by RT-PCR for *Pdr2* |
| rtPdr5-F/R | AACCAGGGTTGTGCTGTTCCC / CACTGCCTTGGGTTTCCTTCTT | Mutant ID by RT-PCR for *Pdr5* |
| rtMdr1-F/R | AGCGAGGCTCTACCCAAAGG / AGCAGACGGCGAAAGCAACA | Mutant ID by RT-PCR for *Mdr1* |
| rtMrp-F/R | GCCATTTTAGCCCTTCGGTG / GCCACTCGCCACTTTTGACC | Mutant ID by RT-PCR for *Mrp1* |
| sbPdr1-F/R | ACCGCAACGCTGAACCTACT / TCTTCGCTTCCTCCTTGTTAT | Mutant ID by Southern for *Pdr1* |
| sbPdr2-F/R | TGCATCGCTGTCTCGGACTC / CGGTGGCAATGAGATAGACACTT | Mutant ID by Southern for *Pdr2* |
| sbPdr5-F/R | GCTCAACAACGTGCAAGGTTAC / TCCTCGGTCAGCGTCTTATTCT | Mutant ID by Southern for *Pdr5* |
| sbMdr1-F/R | GGTGTTGCTTTCGCCGTCTG / GGGGCGAACGAGAATACGGT | Mutant ID by Southern for *Mdr1* |
| sbMrp1-F/R | AATTGCGACCGACGAACAAG / AGTGTATGGCGATGATGAAAAGC | Mutant ID by Southern for *Mrp1* |

* Underlined regions: restriction enzyme sites. Italicized regions with underline: fragments for gateway exchange.
