# Supplementary figures and images for: Differential Contributions of Five ABC Transporters to Mutidrug Resistance, Antioxidion and Virulence of Beauveria bassiana, an Entomopathogenic Fungus
Source: PLoS One. 2013 Apr 15;8(4):e62179. doi: 10.1371/journal.pone.0062179 (PMC3626590; doi:10.1371/journal.pone.0062179)

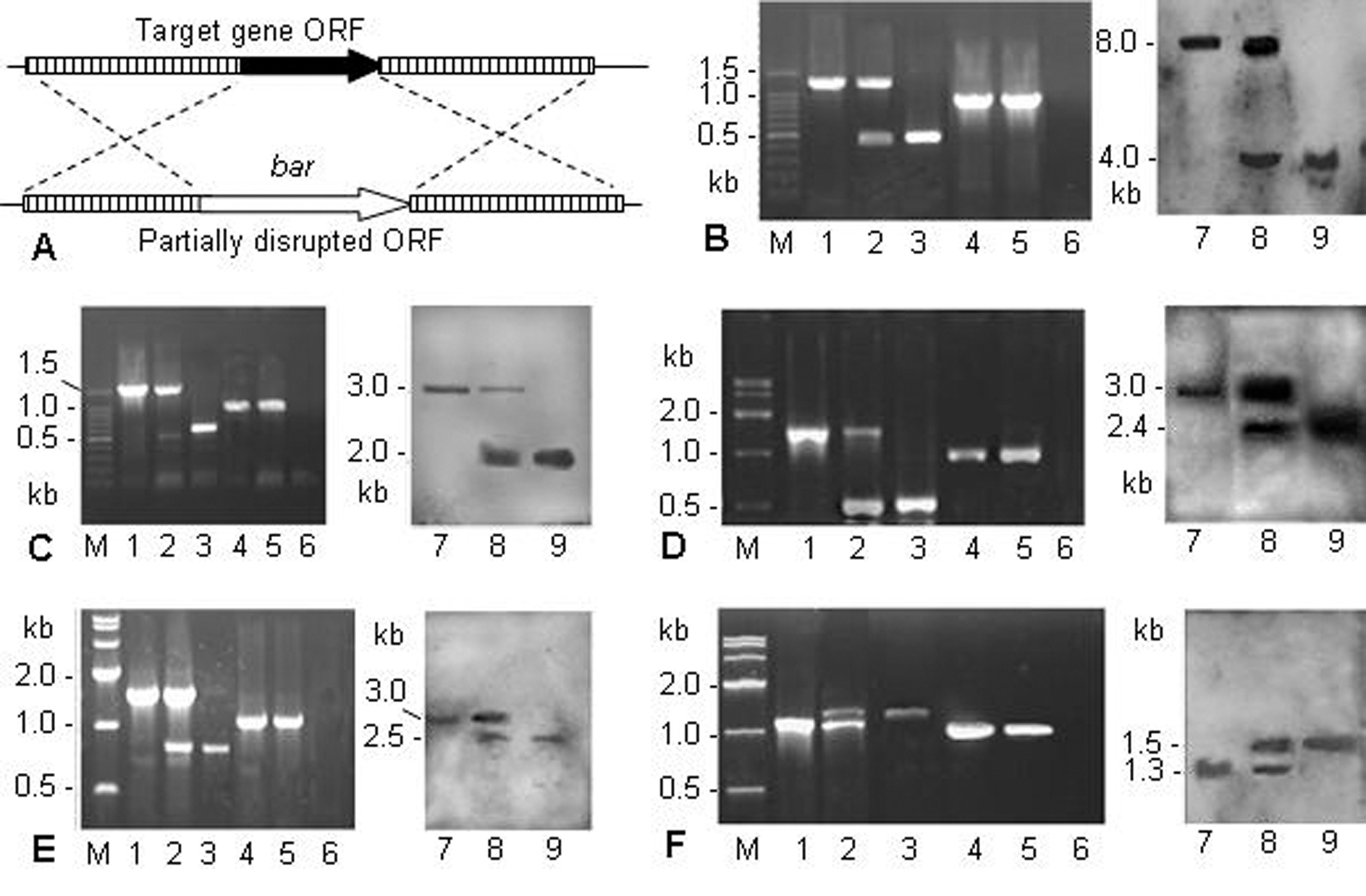

Supplement: Figure S1 — Disruption and complementation of five selected ABC transporter genes in B. bassiana wild-type strain (Bb2860). (JPG) [file pone.0062179.s003.jpg]
